# Exploring New Therapeutic Avenues for Ophthalmic Disorders: Glaucoma-Related Molecular Docking Evaluation and Bibliometric Analysis for Improved Management of Ocular Diseases

**DOI:** 10.3390/bioengineering10080983

**Published:** 2023-08-20

**Authors:** Flaviu Bodea, Simona Gabriela Bungau, Andrei Paul Negru, Ada Radu, Alexandra Georgiana Tarce, Delia Mirela Tit, Alexa Florina Bungau, Cristian Bustea, Tapan Behl, Andrei-Flavius Radu

**Affiliations:** 1 Doctoral School of Biomedical Sciences, University of Oradea, 410087 Oradea, Romania; dr.flaviu.bodea@gmail.com (F.B.); negrupaul59@gmail.com (A.P.N.); dtit@uoradea.ro (D.M.T.); pradaalexaflorina@gmail.com (A.F.B.); andreiflavius.radu@uoradea.ro (A.-F.R.); 2 Department of Pharmacy, Faculty of Medicine and Pharmacy, University of Oradea, 410028 Oradea, Romania; 3 Department of Preclinical Disciplines, Faculty of Medicine and Pharmacy, University of Oradea, 410073 Oradea, Romania; 4 Ducfarm Pharmacy, 410514 Oradea, Romania; adaroman96@gmail.com; 5 Medicine Program of Study, Faculty of Medicine and Pharmacy, University of Oradea, 410073 Oradea, Romania; tarce_alexandra@yahoo.com; 6 Department of Surgery, Oradea County Emergency Clinical Hospital, 410169 Oradea, Romania; 7 School of Health Sciences &Technology, University of Petroleum and Energy Studies, Dehradun 248007, India; tapanbehl31@gmail.com

**Keywords:** glaucoma, molecular docking, retinal diseases, bibliometric analysis, ocular disease therapy, micropulse laser therapy

## Abstract

Ophthalmic disorders consist of a broad spectrum of ailments that impact the structures and functions of the eye. Due to the crucial function of the retina in the vision process, the management of eye ailments is of the utmost importance, but several unmet needs have been identified in terms of the outcome measures in clinical trials, more proven minimally invasive glaucoma surgery, and a lack of comprehensive bibliometric assessments, among others. The current evaluation seeks to fulfill several of these unmet needs via a dual approach consisting of a molecular docking analysis based on the potential of ripasudil and fasudil to inhibit Rho-associated protein kinases (ROCKs), virtual screening of ligands, and pharmacokinetic predictions, emphasizing the identification of new compounds potentially active in the management of glaucoma, and a comprehensive bibliometric analysis of the most recent publications indexed in the Web of Science evaluating the management of several of the most common eye conditions. This method resulted in the finding of ligands (i.e., ZINC000000022706 with the most elevated binding potential for ROCK1 and ZINC000034800307 in the case of ROCK2) that are not presently utilized in any therapeutic regimen but may represent a future option to be successfully applied in the therapeutic scheme of glaucoma following further comprehensive testing validations. In addition, this research also analyzed multiple papers listed in the Web of Science collection of databases via the VOSviewer application to deliver, through descriptive analysis of the results, an in-depth overview of publications contributing to the present level of comprehension in therapeutic approaches to ocular diseases in terms of scientific impact, citation analyses, most productive authors, journals, and countries, as well as collaborative networks. Based on the molecular docking study’s preliminary findings, the most promising candidates must be thoroughly studied to determine their efficacy and risk profiles. Bibliometric analysis may also help researchers set targets to improve ocular disease outcomes.

## 1. Introduction

The anterior section of the human eye is solely responsible for focusing a sharp image of the visual world onto the retina. The primary symptom of retinal disorders is visual impairment, which is not localized and may result from an abnormality at any point along the visual pathway, such as optical aberrations, functional visual impairment, or cortical disorders [1]. 

Central serous chorioretinopathy, age-related macular degeneration, diabetic macular edema, diabetic retinopathy, glaucoma, and retinal vein occlusion are among the most prevalent eye disorders in which inflammatory processes and oxidative stress are essential causative factors [2,3]. Retinal and choroidal vascular disorders constitute the most common causes of visual impairment. The approximate worldwide prevalence of age-related macular degeneration was 196 million in 2020, and due to the aging of the global population, 288 million are anticipated for 2040 [4]. In 2012, the worldwide prevalence of diabetic macular edema was 21 million and of diabetic retinopathy was 93 million. In addition, retinal vein occlusion was the second most common retinal vascular condition, with a worldwide estimated prevalence of 16.4 million individuals in 2008, and an important percentage of those suffering from it developed macular edema [5]. According to studies, central serous chorioretinopathy develops approximately six times more often in men than in women, with an incidence rate of 10 per 100,000 men per year [6]. Moreover, 57.5 million individuals in the world have primary open-angle glaucoma. Individuals over the age of 60, persons with a family history of glaucoma, patients under treatment with glucocorticoids, diabetics, those with high myopia, elevated blood pressure, and a central corneal thickness of less than 5 mm, as well as those who have sustained an eye injury, have an elevated risk of developing glaucoma. Glaucoma is anticipated to impact around 112 million individuals by 2040 [7].

Each of the aforementioned diseases has particular signs and mechanisms of disease development, but they all share the same effect on the retina’s structure and functionality. Given the essential role of the retina in the vision process, the treatment of eye conditions is of the utmost importance. Successful approaches to improving the management of these conditions seek to maintain or recover visual function, ameliorate symptoms, limit the condition’s progression, and improve sufferers’ standard of living [8,9].

Due to its complexity, glaucoma is one of the most studied pathologies in the ophthalmologic field. Glaucoma represents an ensemble of degenerative eye conditions characterized by damage to the optic nerve that, if neglected, can lead to permanent visual deterioration. A disturbance in the production and outflow of aqueous humor causes high intraocular pressure (IOP), which is the primary risk indicator for glaucoma [10].

To preserve vision, glaucoma treatments seek to reduce IOP and slow the progression of the disease. The most common treatment is the application of topical eye medications that either decrease or enhance aqueous humor discharge. Beta-blockers, inhibitors of carbonic anhydrase, and alpha-agonists are among the compounds with beneficial pharmacological action in glaucoma. In certain instances, oral drugs may be recommended for IOP reduction [11].

In conjunction with medication, laser therapy can be utilized to increase or decrease the production of aqueous humor. Argon laser trabeculoplasty (ALT) and selective laser trabeculoplasty (SLT) are widespread procedures that enhance fluid drainage by targeting the trabecular meshwork [12]. Furthermore, micropulse laser therapy (MLT) has emerged as a promising treatment approach for glaucoma. This novel strategy offers several benefits for the treatment of glaucoma. Reducing thermal damage to adjacent tissues is one of the primary benefits. By delivering laser energy in micropulses, MLT enables precise targeting of the trabecular meshwork and additional structures associated with aqueous outflow without causing substantial thermal damage. This not only reduces the possibility of complications but also improves the patient’s comfort throughout the process. MLT’s capacity to accomplish a more uniform distribution of energy is an additional advantage [13].

Surgical procedures may be required when drugs and laser therapy are inadequate to control IOP. Among the surgical techniques accessible are trabeculectomy, a procedure that constructs a new drainage channel, and minimally invasive glaucoma surgery [11].

Rho kinases (ROCKs) have been identified as essential proteins in glaucoma pathogenesis. ROCK1 and ROCK2 are involved in multiple cellular processes, such as the modulation of cell contractility, cytoskeletal structure, and cell adhesion. In glaucoma, their improper functioning has been linked to the development of elevated IOP and consequent optic nerve injury [14].

The ROCK signaling pathway is triggered in the trabecular meshwork, an essential tissue that influences the discharge of aqueous humor. Furthermore, elevated ROCK activity disrupts the actin cytoskeleton, leading to reduced aqueous humor drainage and increased IOP. In addition, it has been demonstrated that ROCKs induce inflammation, fibrotic alterations, and oxidative stress in the trabecular meshwork, thus leading to the progression of the disease [15].

Ripasudil and fasudil, both significant ROCK inhibitors, have emerged as prospective therapeutic options for the treatment of glaucoma. These pharmacological compounds demonstrated their beneficial impact by blocking the activity of ROCKs, resulting in trabecular meshwork rest, enhanced discharge of aqueous humor, and a reduction in IOP [16,17].

Given the implications of the ROCK pathway in glaucoma pathophysiology and the identification of unmet needs in current glaucoma management, in particular the need for better outcome measures in clinical trials [18], the present study was designed to take a dual approach. Starting from two compounds with known and proven activity (i.e., IOP lowering, antioxidant activity, wound-healing activity, increasing drainage from the eye) [16,17], an in silico examination was designed to evaluate the binding potential of compounds without current medical applications to ROCK1 and 2, with the objective of discovering potential uses for new compounds that will require further validation and endorsement studies. The second section of the research is devoted to a thorough and distinct bibliometric assessment of therapy management for ocular diseases, with emphasis on certain more prevalent ocular pathologies, including glaucoma, but also on laser therapies, in particular micropulse laser therapy, as can be seen in the search algorithm used in Web of Science. By reviewing the recent scientific literature in a distinct manner, publications addressing treatment strategies and therapeutic advances in ocular diseases were assessed. 

The current study provides a two-part investigation aimed at incorporating computational approaches (i.e., molecular coupling analysis, virtual screening of ligands, and estimations of some pharmacokinetic parameters) to identify novel compounds not currently used in medical practice but with potential for future applications due to their high binding affinities and at performing a comprehensive bibliometric analysis for the evaluated field. The molecular docking study was based on ligands with demonstrated biological action in the scientific literature (i.e., ripasudil and fasudil) and targeting key proteins (i.e., ROCK1 and ROCK2) identified as being involved in the modulation of glaucoma pathophysiological mechanisms. 

The improvement brought to existing scientific information relies on the demand to develop novel pharmacological strategies to enhance the care of glaucoma patients who do not respond to the current standard of treatment. Furthermore, the contributions to the field of study generated by the present bibliometric analysis are based on the distinct and comprehensive design with emphasis on therapy, including micropulse laser therapy, a topic less addressed in the literature. The results generated by the software and the interpretation of the data contribute to providing an overview of the most relevant and prolific journals, countries, authors, and organizations. The scientific information provided can be an instrument that saves time for researchers in the pre-publication period, facilitating the identification of significant journals, unmet needs, the status of contemporary comprehension in this domain, and the possibility of opening new inter- and multidisciplinary as well as international collaborative networks.

## 2. Materials and Methods

### 2.1. Ligand Preparation

Ripasudil and fasudil, known Rho kinase inhibitors, were selected for the molecular docking investigations. The structures of ripasudil and fasudil were downloaded in the Simulation Description Format (.SDF) format from the PubChem online collection (https://pubchem.ncbi.nlm.nih.gov/, accessed on 20 June 2023). An essential first step in the ligand preparation process consisted of converting the ligands from the SDF format, which is not compatible with AutoDockTools 1.5.7 (https://vina.scripps.edu/, accessed on 20 June 2023), into the native AutoDockTools format, which is the Protein Data Bank, Partial Charge and Atom Type (PDBQT) format, utilizing the Open Babel GUI 2.3.1 program (https://openbabel.org/docs/current/GUI/GUI.html/, accessed on 20 June 2023) [19,20]. The second step involved importing the ligands (i.e., in PDBQT format) into AutoDockTools, where the necessary charges and rotatable bonds were added.

### 2.2. Protein Preparation

The structures of proteins were retrieved from the Research Collaboratory for Structural Bioinformatics Protein Data Bank (PDB) (https://www.rcsb.org/, accessed on 23 June 2023). Specifically, the following protein molecules with their corresponding PDB identifiers were selected: 2ESM (ROCK1 bound to fasudil) [21] and 7JNT (ROCK2 complexed with a selective inhibitor) [22]. The first step in the protein preparation process for molecular docking consisted of removing the co-crystalized water and the co-crystalized ligands using Molegro Molecular Viewer (http://molexus.io/molegro-molecular-viewer/, accessed on 23 June 2023). In the second stage, the protein was imported into the software AutoDockTools, during which hydrogens with polarity as well as Gasteiger charges were inserted. Finally, we saved the protein in PDBQT format.

### 2.3. Molecular Coupling Assessments

The computational studies dealing with the assessment of molecular couplings were conducted via AutoDockTools, a commonly utilized software application. Discovery Studio Visualizer 4.5 (https://www.3ds.com/products-services/biovia/products/molecular-modeling-simulation/biovia-discovery-studio/visualization/, accessed on 26 June 2023) was utilized to produce the 2D and 3D representations, which offer an in-depth representation of the evaluation outcomes (i.e., the bonds between protein and ligand and the spatial arrangement of docked structures). The grid box size for all molecular docking simulations was preset to 60 × 60 × 60 Ångstroms (Å). This box defines the region where the docking instruments will examine potential arrangements. A verification process involving the re-docking of the native ligand of the researched molecule (i.e., the compound that crystallizes with the molecule) along with the comparison of the docked configuration to the native position was conducted prior to docking the targeted ligands. Moreover, by comparing the native molecule to the re-docked compound, the magnitude of similarities between the atomic coordinates of the two positions is used to calculate the root mean square deviation (RMSD). In general, docking methods that produce docking configurations with RMSD values lower than 2 Å (i.e., lower values imply a better match) are efficient at anticipating ligand poses. As calculated by the AutoDock-Tools 1.5.7 program, the RMSD for all docking configurations of the re-docked native compound was lower than 2 Å in the current study.

An important criterion to evaluate the possible affinities and interaction strengths in this context is the emphasis on intra-ligand and protein binding energies. Although direct comparisons between several compounds may not always be possible using binding energies’ absolute values, a given compound’s relative differences in binding sites to a protein are likely to produce results with greater accuracy. This distinction makes identifying more advantageous binding conformations easier and boosts confidence when choosing good candidates. 

To calculate the binding free energy of the ligand-receptor complex, this scoring function integrates several energy factors, such as van der Waals interactions, hydrogen bonds, electrostatic interactions, and torsional strain. The produced poses are sorted according to how well they are projected to bind the chosen protein. Lower rating values in this ranking algorithm denote more reliable and favorable interactions. While the molecular docking process and scoring functions provide valuable insights into the protein–ligand interaction and identify the most probable binding pose, it is essential to complement these computational results with in vivo and in vitro studies. The results from these experimental approaches help validate the accuracy of the predicted binding poses and provide a comprehensive understanding of the ligand’s interaction with the target protein [23].

### 2.4. In Silico Screening

The primary goal of the virtual screening investigation was to find novel compounds that have potential Rho kinase inhibitor properties. Compounds with chemical structures that are comparable were identified using the SwissSimilarity (http://www.swisssimilarity.ch/, accessed on 27 June 2023) web-based instrument. The newly discovered compounds were extracted from the ZINC online database in .SDF format and reformatted with OpenBabelGUI to a form appropriate for AutoDock Vina assessments (https://vina.scripps.edu/, accessed on 27 June 2023), in which charges have been included and rotational bonds were identified. Furthermore, the following stage of investigation consisted of docking them to the targeted molecules in an attempt to identify compounds with significant binding capacity and with prospects for future integration into thorough in silico, in vitro, and in vivo investigations.

### 2.5. Estimates of Pharmacokinetic Data

The SwissADME (http://www.swissadme.ch/, accessed on 28 June 2023) online tool was utilized for predicting some relevant pharmacokinetic properties of molecules considered to have the greatest potential based on the results of the molecular coupling and virtual screening of ligands examinations.

## 3. Results

### 3.1. Molecular Docking of Compounds against ROCK1

**Figure 1:**
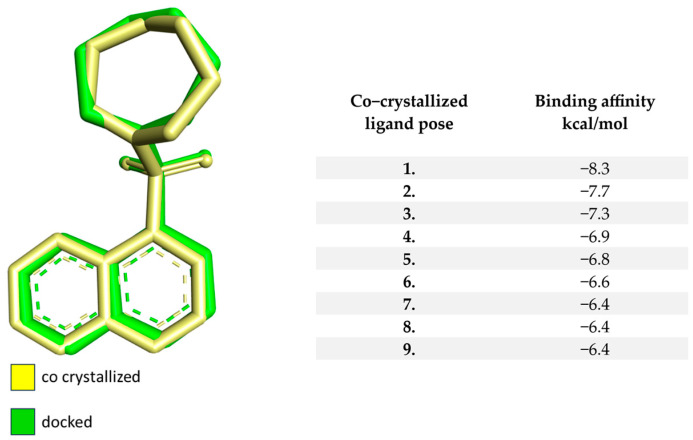
The binding capacity of the re-docked native compound for ROCK1 and the structure of the native ligand overlaid with the re-docked compound.

The preliminary step in the docking procedure is the reattachment of the molecule with which the molecule of protein is co-crystallized. The grid-box dimensions and coordinates are 60 × 60 × 60 Å, and X = 52.29, Y = 99.79, and Z = 28.53, respectively. The RMSD of the re-docked compound compared to the native ligand is 0.88, which fits into the general values accepted by the literature, indicating a good molecular docking algorithm. In the case of 2ESM, the native ligand is represented by fasudil, a potent Rho kinase inhibitor. The best re-docked position of the native ligand has a binding potential in the form of an affinity of −8.3 kcal/mol for the evaluated protein. Figure 1 displays the values of affinity for the protein of the top 9 docked poses and the docked arrangement of fasudil overlaid on the co-crystallized framework.

**Figure 2:**
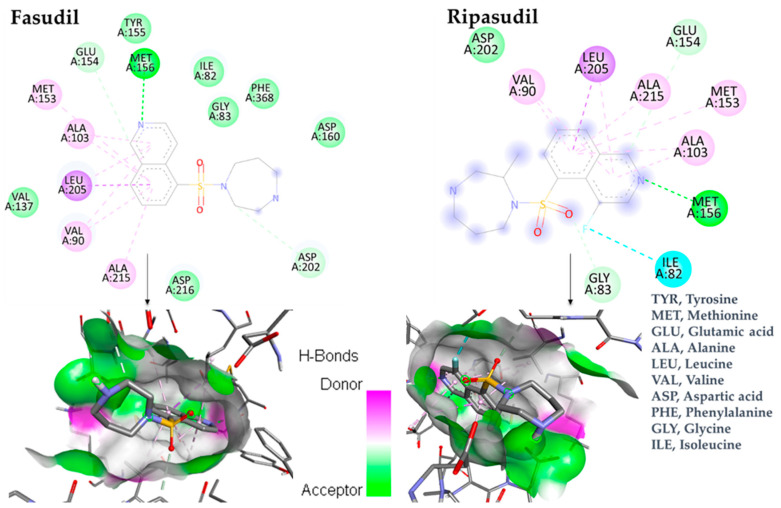
Comprehensive two-dimensional and three-dimensional interactions of ligand–ROCK1.

The docking results in the case of ripasudil concluded that the best docking pose of this compound had a higher affinity (−8.6 kcal/mol) for the protein compared to the native ligand, fasudil (−8.3 kcal/mol). Figure 2 depicts the bonds between specific aminoacids from the protein and the ligands. The illustration contains 2D representations of the interactions and 3D diagrams of the ligands within the protein’s binding pocket.

The two-dimensional representations show that fasudil is involved in interactions with MET156, GLU154, MET153, ALA103, LEU205, VAL90, ALA215, and ASP202. Ripasudil interacts with VAL90, LEU205, ALA215, GLU154, MET153, ALA103, MET156, ILE82, and GLY83. The common amino acids that interact with fasudil and ripasudil include MET156, MET153, ALA103, LEU205, VAL90, ALA215, and GLU154. Additionally, ripasudil interacts with ILE82 through the fluorine atom, which could be responsible for the stronger binding affinity of this compound for ROCK1. 

In the context of our docking study, a compelling interaction in which both compounds form essential hydrogen bonds with MET156, an amino acid residue located within the binding region of the protein. It is essential to acknowledge from the stability of the protein–ligand complex that the creation of these hydrogen bonds plays a crucial part in coordinating the binding mechanism. It is vital that these hydrogen bonds with MET156 form because they increase the stability of the protein–ligand complex. This interaction will likely improve how strongly the compounds bind and how specifically they interact. This emphasizes how vital MET156 is in ensuring the protein complex is stable, which could potentially positively impact its therapeutic benefits.

### 3.2. Molecular Docking of Compounds against ROCK2

**Figure 3:**
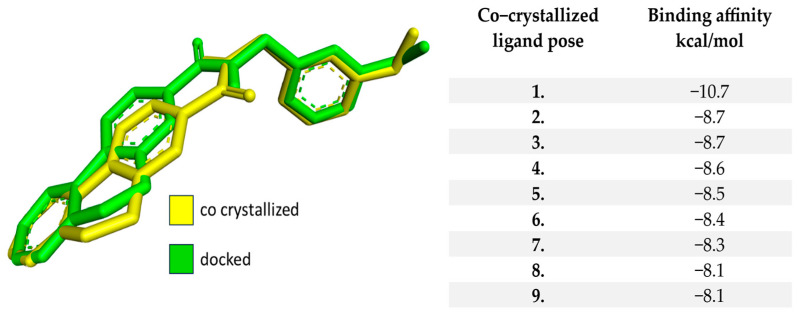
The representation of the most elevated binding affinities of the re-docked native compound for ROCK2 and the structural representation of the native ligand superimposed with the re-docked ligand.

As in the case of ROCK1, the first step was to re-dock the protein’s (ROCK2) co-crystallized ligand, N-[(3-methoxyphenyl) methyl]-5H-[1] benzopyrano[3,4-c]pyridine-8-carboxamide. The grid-box dimensions and coordinates were set at 60 × 60 × 60 Å and X = 47.05, Y = 68.16, and Z = 36.18, respectively. The RMSD value of the re-docked native ligand compared to the co-crystallized was 1.065, a value within the acceptable range stated in the literature. The best position of the re-docked compound presents a binding affinity of −10.7 kcal/mol. Moreover, the affinity values of the top nine docked positions and an overlay representation of the docked and co-crystallized structures are displayed in Figure 3.

**Figure 4:**
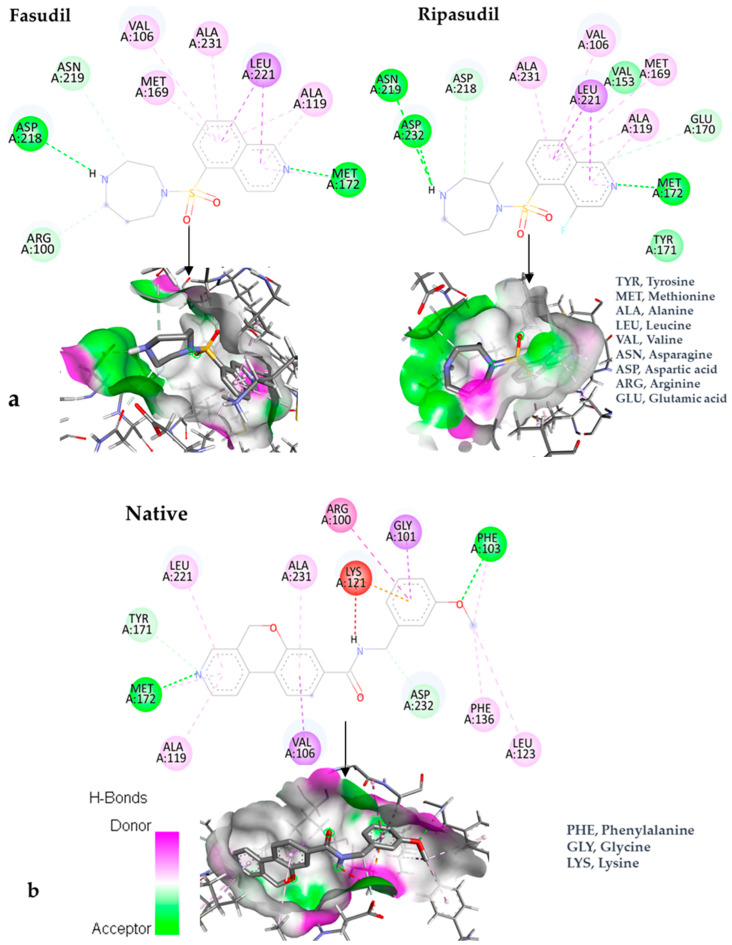
In-depth two-dimensional and three-dimensional interplays of ligand–ROCK1. (**a**) Interactions between different structures of fasudil and ripasudil with the substructures of the target protein; (**b**) Interactions between different structures of the native ligand with the substructures of the target protein.

The docking results in the case of ripasudil concluded that the best docking position of this compound presented a binding potential of −9.1 kcal/mol for the evaluated protein. fasudil had a binding affinity of −8.7 kcal/mol. Ripasudil and fasudil showed lower affinities for the protein than the native ligand. Figure 4 depicts the interactions between the ligands and the protein. The illustration contains two-dimensional representations of the interactions and three-dimensional models of the compounds within the protein’s binding site.

According to the two-dimensional model, the native ligand interrelates to the following amino acids: ASP232, PHE136, LEU123, PHE103, GLY101, ARG100, LYS121, ALA231, LEU221, TYR171, MET172, ALA119, and VAL106. Fasudil interacts with MET172, ALA119, LEU221, ALA231, VAL106, MET169, ASN219, ASP218, and ARG100. Ripasudil interacts with MET172, GLU170, ALA119, LEU221, MET169, VAL106, ALA231, ASP218, ASN219, and ASP232. The common amino acids interacting with fasudil and ripasudil are represented by: MET172, ALA119, LEU221, ALA231, VAL106, and ARG100. As for the native ligand, it has two common amino acids with fasudil and ripasudil, namely, ALA119 and MET172.

During the in-depth analysis of protein–ligand interactions, a notable observation emerges. Although the three compounds’ chemical structures are different from those of the native ligand, they share contact with MET172 via a hydrogen bond. This finding underscores the pivotal role of this amino acid in establishing a stable complex between the protein and the ligand. One of the amino acids that could play an essential role in stabilizing the ligand–protein complex is represented by ASP218, which forms a hydrogen bond in the case of fasudil. Additionally, in the case of ripasudil, interactions with ASN219 and ASP232 are evident. For the native ligand, interaction with PHE103 might also have a crucial role in complex formation.

### 3.3. In Silico Screening of the Candidate with the Most Potential

Among the investigated molecules, ripasudil showed the highest affinity towards both proteins (ROCK1 and ROCK2). Starting from the chemical structure of this compound, a simulated screening of ligands via the SiwssSimilarity online tool has been conducted. The objective of this specific investigation was to discover compounds with comparable chemical structures to ripasudil and determine their affinity for ROCK1 and ROCK2 via molecular docking studies.

SwissSimilarity’s digital platform analyzes and identifies similarities between compounds using a range of chemical fingerprinting methodologies. In these systems, two- and three-dimensional strategies of structural comparison are utilized. Furthermore, in order to seek compounds with similar physicochemical properties, SwissSimilarity provides data regarding the physical and chemical properties of the compounds [24].

To display and distinguish molecular structures, extended-connectivity chemical fingerprints (ECFPs) are commonly used. As circular fingerprints, ECFPs contain information about a molecule’s parts. The ECFP algorithm generates these fingerprints using a graph-based methodology by analyzing all possible paths across atoms in a molecular structure of a particular dimension. It has been demonstrated that ECFPs are widely utilized in a variety of applications, such as virtual screening, compound library clustering, and similarity research [25]. The screening method was set as ECFP, and the search was performed on the ZINC (Lead-like) database (https://zinc.docking.org/, accessed on 20 April 2023). From this search, twenty molecules presenting similarity values ranging between 0.722 and 0.475 were identified. Furthermore, a higher similarity score indicates a more significant similarity to the parent substance.

Table 1 provides the scores of similarities, chemical structures, and affinities of the five most prospective molecules, as determined by molecular docking evaluation, with regard to their affinity to ROCK1.

ZINC000000022706 showed the most elevated affinity (−9.0 kcal/mol) for ROCK1, surpassing the parent molecule ripasudil (−8.6 kcal/mol) and the native ligand fasudil (−8.3 kcal/mol).

Ripasudil was kept as the parent compound in the screening process for ROCK2 because it has a higher affinity for the protein compared to fasudil. Table 2 provides the scores of similarities, chemical structures, and affinities of the five most prospective molecules, as determined by molecular docking evaluation, with regard to their affinity to ROCK2.

ZINC000034800307 showed a higher affinity (−8.8 kcal/mol) for the protein than fasudil (−8.7 kcal/mol) but a lower affinity than ripasudil (−9.1 kcal/mol) and the native ligand (−10.7 kcal/mol). The observed values are also consistent with the average values found in published research evaluating the binding potential of various compounds to ROCK2 (i.e., −7.39 to −9.07 kcal/mol) [26].

**Figure 5:**
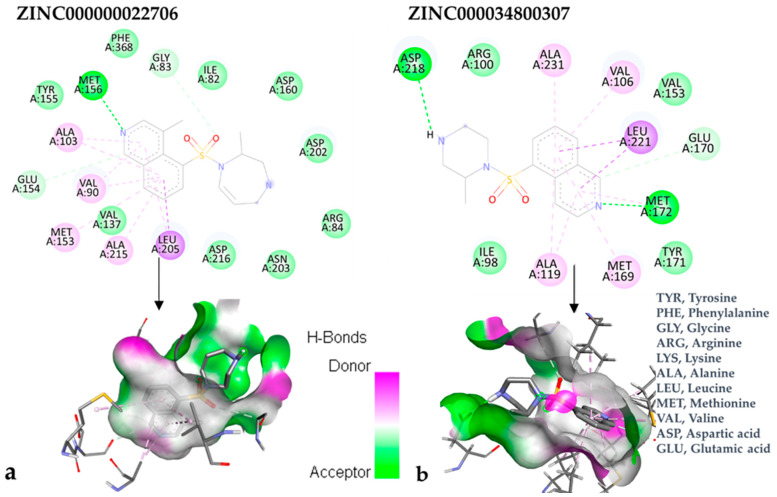
Comprehensive two-dimensional and three-dimensional interactions of ligand–target protein. (**a**) Interactions with ROCK1; (**b**) interactions with ROCK2.

Figure 5 depicts the interactions between the identified ligands and the target protein. The illustration contains two-dimensional representations of the ligands and three-dimensional models of the compounds within the protein’s binding site.

In accordance with the two-dimensional representation, ZINC000000022706 interacts with the following ROCK1 amino acids: GLY83, MET156, ALA103, GLU154, VAL90, MET153, ALA215, and LEU205. Both ripasudil (parent compound) and ZINC000000022706 interact with VAL90, LEU205, ALA215, GLU154, MET153, ALA103, and MET156, indicating a similar binding process to ROCK1. Even though the parent compound additionally interacts with ILE82, the binding affinity of ZINC000000022706 is higher, indicating a more stable ligand–protein complex. A similar binding mechanism was revealed when we examined the ZINC000000022706 protein’s interaction with fasudil in greater detail. Specifically, most interactions occur through the isoquinoline ring, displaying a high resemblance. An exception to this pattern is observed with GLY83, which establishes its binding to ZINC000000022706 via a sulfonyl group. Consequently, considering the comparable binding energies, the significant number of shared amino acids, and the chemically akin structure of ZINC000000022706 compared to fasudil, this substance emerges as a plausible candidate with therapeutic potential. Given the elevated number of shared amino acids, it is reasonable to assume that ZINC000000022706 could participate in comparable molecular recognition processes, perhaps targeting similar biological pathways as fasudil. The alignment of binding energies additionally offers a theoretical framework for assessing the potency of these interactions.

Therefore, evidence of similar binding patterns to the protein as the parent compound is apparent in the case of ZINC000000022706. Notably, the recurrence of hydrogen bond formation with MET156 is a noteworthy observation. This recurrence substantiates the hypothesis that ZINC000000022706 can potentially emerge as a therapeutic candidate characterized by similar attributes to the parent compound.

ZINC000034800307 interacts with the following ROCK2 amino acids: ASP218, ALA231, VAL106, LEU221, GLU170, MET172, MET169, and ALA119. Comparing the ligand–protein interactions for both ZINC000034800307 and the parent compound, ripasudil, we can see that both ZINC000034800307 and ripasudil interact with the following amino acids: ASP218, ALA231, VAL106, LEU221, GLU170, MET172, MET169, and ALA119, indicating a similar binding method to the protein. Ripasudil’s or fasudil’s interactions with ROCK2 and those with ROCK1 are similar, suggesting a possible inference even if the parent chemical of ROCK2 is not included among the known medications with established effects on the pathophysiology under study. A similar binding pattern is seen when the interaction mode between the parent compound–protein and ZINC000034800307–protein is examined. This resemblance encompasses both the chemical groups and the interacting amino acids. Notably, the isoquinoline ring mediates the majority of interactions with the protein. There are a few exceptions, though: ripasudil interacts with ASP232, ASN219, and ASP218 through the 1,4-diazepane ring in this scenario.

In contrast, contact occurs through a piperazine ring in the instance of ZINC000034800307 instead of a 1,4-diazepane ring. The isoquinoline-mediated interactions have a recurring motif, highlighting a consistent and possibly meaningful protein binding method. The distinctive interactions with particular amino acids, such as ASP232, ASN219, and ASP218 in the case of ripasudil, and the piperazine ring in the case of ZINC000034800307, point to structural modifications that may be responsible for the selectivity and affinity of these interactions. Moreover, the comparable binding patterns and shared structural elements between the interactions of ZINC000034800307–protein and the parent compound–protein highlight the potential of ZINC000034800307 as a promising candidate for further investigation. Its interaction similarities, especially the mode of interaction with protein residues and functional groups, underscore the potential for targeted bioactivity, warranting deeper exploration for potential therapeutic implications.

A parallel trend becomes apparent with ZINC000034800307, mirroring the findings elucidated by ZINC000000022706. Precisely, a reminiscent mode of interaction with MET172 emerges. A hydrogen bond ensues between the ligand and this specific amino acid, substantiating its instrumental function in bolstering the stability of the ligand–protein complex.

### 3.4. Computational Assessments of Relevant Pharmacokinetic Data for Newly Found Molecules

By utilizing SwissADME, important pharmacokinetic data for two possible Rho kinase inhibitors has been evaluated. The findings of the computational ADME assessment are outlined in Table 3.

Ripasudil has a molecular weight of 359.8 g/mol and fasudil has a molecular weight of 291.37 g/mol, while the investigated compounds have a molecular weight of 319.42 g/mol and 291.37 g/mol. Furthermore, the investigated compounds also have under five H-bond donors (one for both), under ten H-bond acceptors (five for both), and Log P values of 2.50 (ZINC000000022706) and 2.10 (ZINC000034800307), which fall under Lipinski’s list of five concepts [27], suggesting the fact that the identified molecules have good oral bioavailability and drug-like characteristics. ZINC000000022706 is a CYP2D6 and CYP3A4 inhibitor, suggesting that it has the potential to alter drug metabolism and interactions with other compounds processed by these enzymes. ZINC000034800307 does not affect the investigated enzymes.

Utilizing digital models and algorithms, SwissADME can assess permeability, solubility, pharmacokinetics, and lipophilicity. Computational approaches to assessing the ADME characteristics of a compound are beneficial to preliminary drug design, but they cannot replace in vivo assays. In contrast, they seek to develop a rapid and cost-effective approach to assessing the pharmacokinetic characteristics of small molecules [28].

The integration of advanced computational methodologies, including molecular docking, ligand-based virtual screening, and ADME evaluation, within the realm of glaucoma research offers promising avenues for revolutionizing patient care and augmenting therapeutic options in clinical practice. These computational tools hold the potential to yield profound insights into the intricate landscape of drug discovery and development, thereby fostering the emergence of more efficacious and tailored treatment strategies for individuals afflicted by glaucoma.

Molecular docking, for instance, empowers researchers to predict the binding affinity and conformational preferences of potential therapeutic agents directed toward specific protein targets, as exemplified by the case of ROCK1 and ROCK2 in glaucoma. Such predictive capabilities facilitate the identification of novel molecular entities that exhibit a heightened propensity to engage with these targets and elicit favorable modulatory effects upon their biological functions.

Ligand-based virtual screening, tailored to the context of glaucoma, serves to substantially broaden the array of candidate compounds warranting consideration. Through an analytical lens rooted in structural and chemical similarity to established active agents, this methodology effectively enlarges the repertoire of potential drug candidates, thus augmenting the likelihood of discovering efficacious treatment regimens.

The judicious inclusion of ADME evaluations at the outset of the drug discovery journey endows researchers with the capacity to discern and prioritize compounds with enhanced potential for favorable outcomes within clinical trials and eventual real-world application. This anticipatory consideration of pharmacokinetic characteristics guides the selection of candidates that hold promise for efficacious therapeutic interventions. Furthermore, the integration of computational techniques, such as molecular docking and virtual screening, offers the advantageous prospect of diminishing the reliance on early stage animal testing.

The subsequent characteristics reflect the research’s strengths: estimates of the binding capacity and the mechanism of interaction between a protein of interest and the ligand with documented biological impacts, which can be used to guide the design of novel ligands; evaluation of a large database of molecules for possible lead compounds; and a comprehensive analysis of the physicochemical and pharmacokinetic properties of newly discovered molecules.

## 4. Bibliometric Analysis

Bibliometric studies are essential in gaining a comprehensive understanding of publication trends, assessing the impact of the research, and understanding the research landscape in a specific field. While the selection of the search algorithm is of utmost importance, the choice of the source database is also crucial. The Web of Science (W.o.S) collection of databases has been used for the present investigation due to its comprehensive accumulation of documents across multiple fields, citation indexing capabilities, and global coverage. The following search algorithm was used to identify relevant articles related to therapy and micropulse in the field of ophthalmology: ALL = (ophthalmology OR retinal diseases OR Diabetic macular edema* OR Retinal vein occlusion OR Glaucoma OR Central serous chorioretinopathy OR Age related macular degeneration) AND ALL = (therapy OR micropulse). A total of 43,450 documents were identified, of which 31,301 (70.04%) were articles, 5927 (13.64%) were review articles, 3643 (8.38%) were meeting abstracts, and 2524 (5.81%) were proceeding papers. The remaining document types had fewer than 1000 classified documents each.

Regarding the language distribution of the identified documents, we found that English is the most commonly used language, accounting for 95.94% (41,685) of the total papers. German was the second most common language, accounting for 2.64% (1149) of the complete paper count. The percentages for French and Portuguese were 0.757% (329) and 0.168% (73). The other languages had fewer than 50 documents.

**Figure 6:**
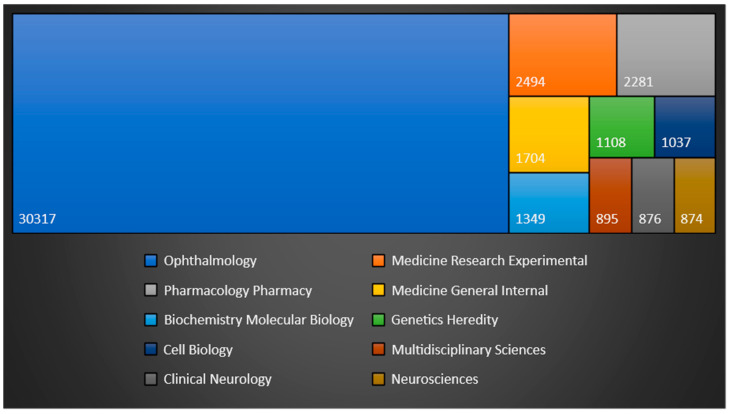
Treemap visualization of the Top 10 Categories.

The identified documents were classified into 165 W.o.S categories, with the critical caveat that a single manuscript might be allocated to more than one category. The following categories had the highest number of assigned manuscripts: “Ophthalmology” 30,317 documents; “Medicine Research Experimental” 2494; “Pharmacology Pharmacy” 2281; “Medicine General Internal” 1704; “Biochemistry Molecular Biology” 1349; “Genetics Heredity” 1108; and “Cell Biology” 1037; other categories had under 1000 manuscripts assigned to them. Figure 6 represents the tree map of the top 10 most populated categories according to W.o.S.

Only English-language articles were considered for the present study, limiting the number of papers assessed to 29,882. The analysis was performed using VOSviewer version 1.6.19 [29,30] and the built-in analysis tools available within the W.o.S system. Furthermore, the necessary information has been extracted from W.o.S as tab-delimited files encompassing the complete record and cited documents’ references using the Export function.

The first paper indexed in W.o.S collection of databases matching the search algorithm was published in 1945. For a more comprehensive approach targeting novel elements in the management of ocular diseases (i.e., micropulse laser therapy), the years 2011–2023 were chosen as the period of bibliometric evaluation and science mapping research. For the evaluated time period, we determined the most prolific nations, journals, authors, articles, and organizations in the field under consideration.

The country collaboration system diagram was generated using VOSviewer to determine the collaborative relationships between countries. The size of each individual bubble is determined by the number of published articles. The width of the band connecting two nations is directly related to the collaboration between those countries, while the color of each bubble is determined by the cluster in which the country was categorized. Countries that often publish articles together are usually classified in the same cluster.

The median publication year and citation mapping of the journals examined were determined as well. In the journal’s median publication year diagram, the color of each sphere reveals the average publication year, and the size of the sphere is directly related to the overall number of papers published in that journal. The bubbles in the map are color-coded from dark blue to yellow. Darker colors represent an early publication year, while lighter colors, specifically yellow, indicate a later publication year. This color gradient visually represents the time distribution of the articles on the map. To improve precision, the years are represented fractionally (e.g., 2005.50 indicates the midpoint of 2005). In the context of the citation system diagram, the size of the sphere is directly correlated with the total number of published papers, the sphere color shows the cluster, and journals that frequently cite one another are typically grouped together. The width of the band connecting two journals is proportional to the frequency of citations between them.

Lastly, the keyword co-occurrence network and the keyword bubble maps were generated for each period. The hue of the keyword sphere diagram shows the mean number of citations that an item containing the keyword has obtained. Moreover, darker hues indicate fewer citations, whereas lighter hues, particularly yellow, indicate more citations. The magnitude of each sphere indicates its frequency of occurrence. In the keyword co-occurrence system diagram, the dimension of the spheres indicates the number of occurrences, the width of the band linking two words is directly related to the number of co-occurrences, and the color reveals the cluster, with frequently occurring keywords typically clustered together.

### 4.1. Period 2011–2023

#### 4.1.1. Assessment of the Most Prolific Nations

During the period under examination, the overall number of nations contributing to scientific output rose from 103 to 151, indicating the growing interest of more countries in this field. The United States remains the most significant contributor, with 6736 (35.02%) published papers. The average citation/article published by the United States is 26.66, indicating that these articles had a significant impact on the field. China occupies second place in regard to the number of papers that have been published (2503, 13.01%), and it has an average citation/article of 12.71. Ranked third is England, with 1555 (8.09%) published documents and an average citation/article of 30.28, indicating the high impact of these articles. Out of the top-ranked countries, France stands out with the highest average citation/article (32.62). Table 4 lists the top ten nations in terms of publication prolificacy in the discipline assessed from 2011 to 2023.

#### 4.1.2. Evaluation of the Most Productive Journals

A total of 1952 journals published documents that fit the search parameters between 2011 and 2023. The most productive journal of this period is *Investigative Ophthalmology & Visual Science*, with a total of 896 (4.66%) published documents. Ranked second is *Retina—The Journal of Retinal and Vitreous Diseases*, with a total of 799 (4.15%) published documents, and ranked third is *Graefe’s Archive for Clinical and Experimental Ophthalmology*, with 615 (3.20%) published documents. The journal *Ophthalmology* stands out with the highest total citations received (compared to the other journals included in the top 10), 39,344, and also with the highest average citation/article (66.24). Table 5 shows some of the most prolific journals that published papers between 2011 and 2023.

#### 4.1.3. Assessment of the Most Prolific Authors

During the evaluated period, 60,211 authors significantly supported scientific advancement. Bandello F., affiliated with Vita-Salute San Raffaele University, Italy, is the most productive author of this period, with 112 published documents. Ranked second is Hauswirth WW, with 92 published documents, affiliated with the University of Florida, United States. Holz, F.G., has the highest average citation per document out of the authors listed among the first ten, receiving a total of 4809 citations for the 73 published documents, resulting in an average citation per document of 65.88. The ten most prolific authors of the evaluated period are listed in Table 6.

#### 4.1.4. Citation Analysis

The number of published articles increased from 10,649 (1945–2010) to 19,233 (2011–2023). The article that had the most citations during this period was published by Sawcer, S., in 2011 and titled “Genetic risk and a primary role for cell-mediated immune mechanisms in multiple sclerosis” in the journal *Nature*, which has an impressive IF of 69.504. Ranked second in terms of citations is the article titled “Intravitreal Aflibercept (VEGF Trap-Eye) in Wet Age-related Macular Degeneration”, published by Heier, J.S., in the journal *Ophthalmology* in 2012. Table 7 presents the most cited articles of this period.

#### 4.1.5. The Topic’s Most Involved Organizations

The count of active organizations rose from 4474 in 2011 to 11,369 during the current period, indicating the increase in attention that this subject receives. The University of California System remains the most active organization, with a total of 885 (4.60%) publications. Ranked second is the University of London with 812 published documents, which is closely followed by University College London with 749 publications. Table 8 presents the most productive organizations during the 2011–2023 period.

### 4.2. Science Mapping

#### 4.2.1. Networks of Collaboration between Nations

**Figure 7:**
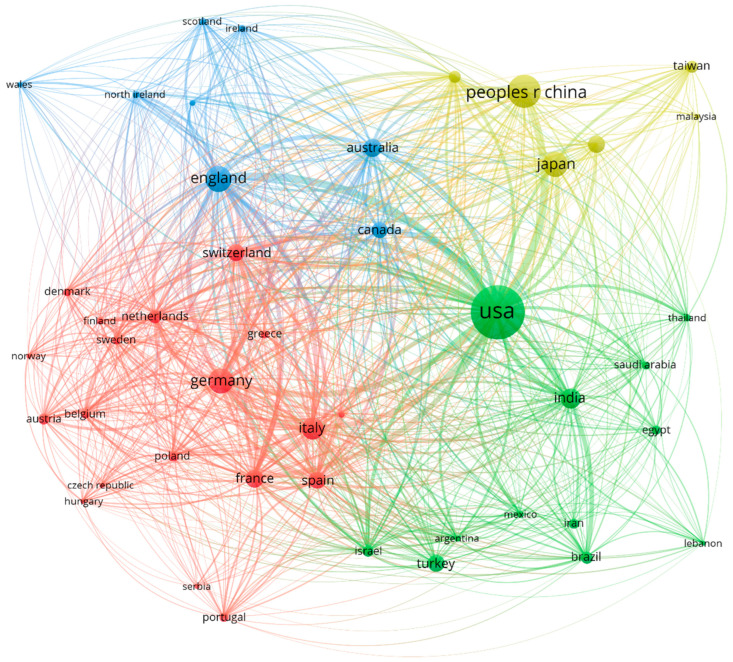
Collaborative writing networks between countries for the period under review, revealed by VOSviewer.

Figure 7 is an interconnected diagram displaying collaboration paths between nations. For a country to be represented in the network map, a minimum requirement of 50 published papers was imposed, thus guaranteeing a robust dataset, resulting in the inclusion of 45 nations that met this condition. These nations were separated into four distinct groups. The red cluster consists of 19 nations, led by Germany, based on published papers. Furthermore, this cluster mainly contains countries from the European continent, indicating a strong collaborative relationship between countries located in Europe. The green cluster consists of 12 countries, and the United States leads based on the papers that have been published. The blue cluster consists of eight nations, led by England. Furthermore, the yellow cluster includes six countries, and China leads in terms of published documents. Strong collaborative relationships form between the United States and the following countries: China (458), England (445), Germany (281), Canada (228), and Italy (214). A strong collaborative relationship was also formed between England and the following countries: Germany (214), Australia (145), Italy (144), and France (143).

#### 4.2.2. Resource Average Publication Year and Citation System Diagram

**Figure 8:**
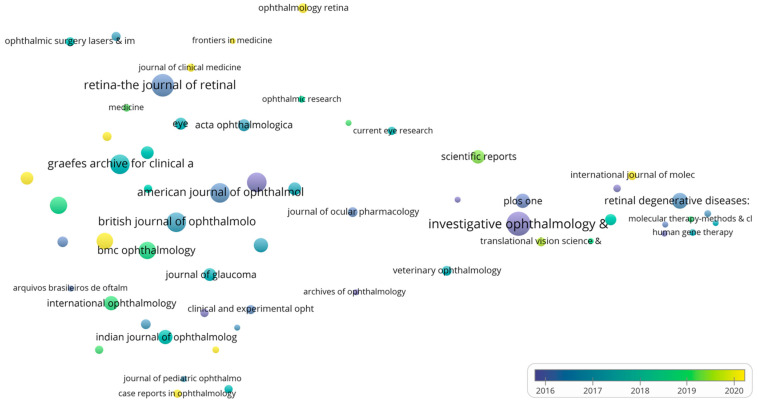
Average journal publication year 2011–2023 (VOSviewer).

Figure 8 depicts a node diagram showcasing the mean year of publication in the case of journals that have published a minimum of 50 articles. *Investigative Ophthalmology & Visual Science*, the most prolific scientific periodical of this time period, possesses an average publication year of 2015.53, indicating that during the 2011–2023 period, documents were published steadily without a substantial increase near the end of the time frame. *Retina—The Journal of Retinal and Vitreous Diseases* ranked second based on the papers that have been published and possesses a mean publication year of 2016.41, whereas *Graefe’s Archive for Clinical and Experimental Ophthalmology*, which is the third most prolific scientific periodical of this time frame, encounters an average publication year of 2017.47, suggesting that more papers have been released at the termination of the period. The majority of the period’s papers were published towards the completion of the period in subsequent journals: *Ophthalmology and Therapy* (2021.25), *Clinical Ophthalmology* (2020.27), *Journal of Clinical Medicine* (2021.10), *Frontiers in Medicine* (2021.71), *Ophthalmology Retina* (2020.25), and *International Journal of Molecular Sciences* (2020.90).

**Figure 9:**
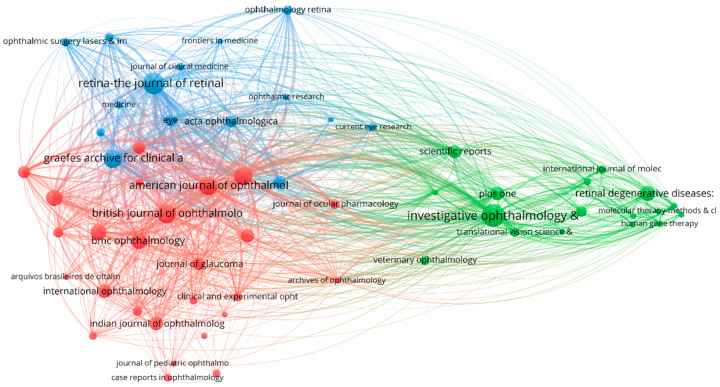
2011–2023 resource citation system map utilizing VOSviewer.

Figure 9 depicts a diagram of source citations. The journal requirements for inclusion remained unchanged from the previous figure. The scientific periodicals are organized into three separate clusters, with the red cluster containing 26 journals and being led on the basis of published documents by the *British Journal of Ophthalmology*. *Investigative Ophthalmology & Visual Science*, the most influential journal of this time frame, leads the group of 17 journals included in the green cluster. Furthermore, the blue cluster consists of 15 scientific journals and is led by the period’s second-highest-producing journal, *Retina—The Journal of Retinal and Vitreous Diseases*. The red cluster and the blue cluster are closely intertwined, indicating that the subjects found in journals included in these clusters are closely related, and they frequently cite each other. To further validate this fact, on a closer analysis of the network map, we can notice that articles from *Retina—The Journal of Retinal and Vitreous Diseases* were often cited by articles from *Ophthalmology* (link strength: 994), the *American Journal of Ophthalmology* (847), and *Graefe’s Archive for Clinical and Experimental Ophthalmology*.

#### 4.2.3. Keyword System Mapping and Terms Co-Occurrence System Diagram

**Figure 10:**
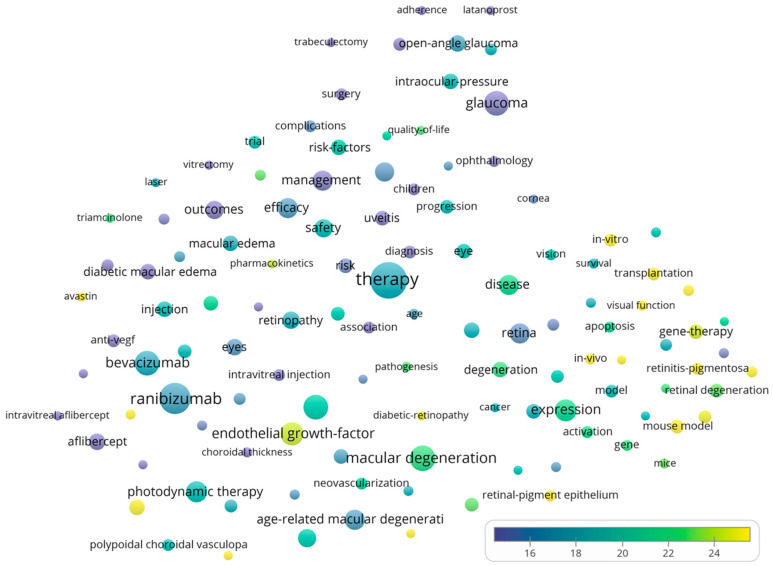
Map of items used highly frequently in the field.

Figure 10 depicts the bubble map of extremely regular terms utilized for searches in the field in the period 2011–2023. Only words with a minimum occurrence of 200 are represented in the figure. The following words had a high occurrence: “therapy (3279 occurrences, 17.92 average citations/document)”, “ranibizumab (2327, 17.27)”, “macular degeneration (1651, 21.79)”, “bevacizumab (1503, 17.75)”, “glaucoma (1478, 12.72)”, and “optical coherence therapy (1477, 20.21)”. Terms that had high average citations/document are represented by: “indocyanine green angiography (209, 34.35)”, “mouse model (457, 31.56)”, “geographic atrophy (236, 30.22)”, “in vivo (308, 29.99)”, “differentiation (309, 29.78)”, “Avastin (211, 28.20)”, and “intravitreal ranibizumab (250, 25.31)”.

**Figure 11:**
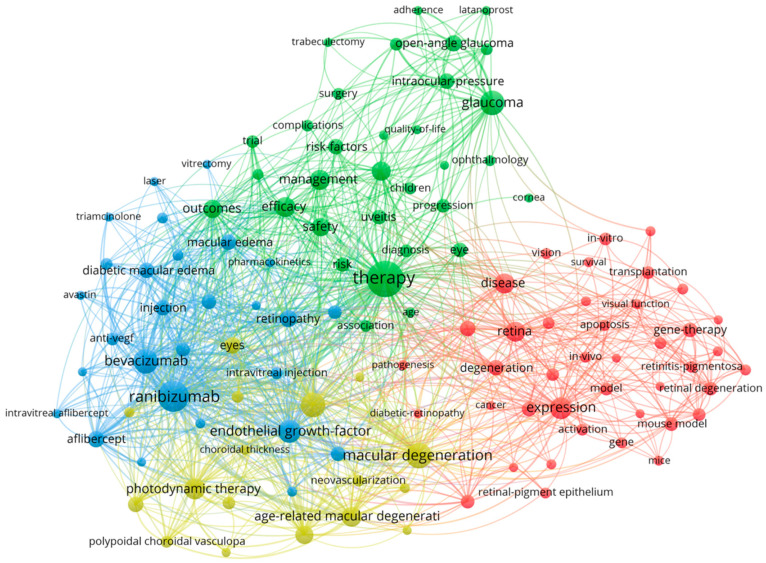
High-frequency term co-occurrence network map.

Figure 11 depicts the network map of keyword co-occurrence. The inclusion criteria for the terms were kept unmodified from the previous figure. A total of four clusters are formed. The red cluster includes 41 terms that are mainly focused on treatment approaches, retinal health, and various diseases. The green sphere contains 32 terms associated with glaucoma medical management and therapy. Moreover, 28 keywords are contained in the blue cluster. This cluster appears to focus on the topic of treatment approaches and conditions related to the retina, particularly anti-vascular endothelial growth factor treatments. The yellow cluster includes 17 terms that are mainly focused on various aspects related to retinal disorders, particularly age-related macular degeneration and associated conditions.

### 4.3. Discussions

The United States was the most prolific nation during the assessed time period, indicating that authors from this country are highly interested in the evaluated topic. In 2010, 50% of the countries in the top 10 were from Europe, while in the period 2011–2023, the number of European countries reduced to 40%. China is worth mentioning as it published only 218 documents until 2010 but experienced a rapid increase in interest in the subject by publishing 2503 articles in the period 2011–present.

A noteworthy observation emerges through an analysis of the co-authorship network map. Starting in 2011, a distinct separation between the clusters became evident, indicating that collaboration networks had solidified during the last decade.

**Figure 12:**
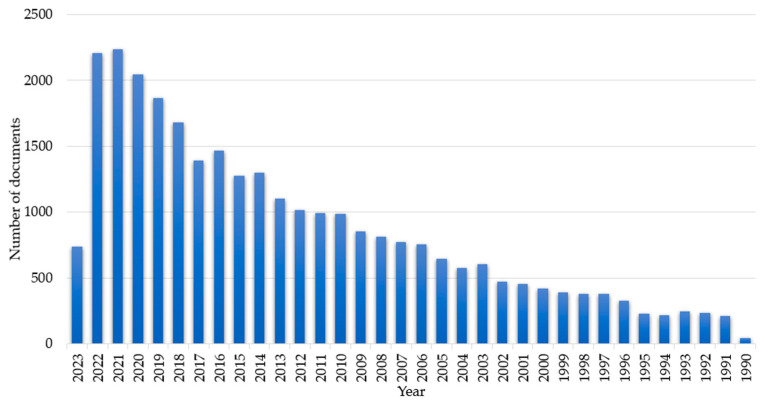
Number of published documents (1990–2023).

Figure 12 shows the total number of papers published annually to illustrate the increasing trend with regard to the number of published papers and researchers’ keenness on this topic. Because the total number of papers released before 1990 was less than 40 per year, the chart covers data from 1990 to 2023, thus providing better clarity. The number of papers published has steadily increased throughout the years, reaching a record in 2021 with 2234 articles published. However, the number of published papers fell slightly in 2022 compared to the previous year, with a total of 2208. This could be the result of a temporary decrease in research productivity or a shift in publication trends. More research would be required to uncover the underlying variables that contributed to this slight decline.

Although authors from countries grouped in the same cluster are more likely to collaborate, we cannot ignore the fact that collaborative relationships are also formed with authors from countries not located in the exact same group. Identifying the most prolific nations in this scientific area of interest and the countries that are most likely to collaborate should provide a solid foundation of knowledge and insights for authors interested in this topic and those looking for potential collaborators.

Overall, the most productive journal is the *American Journal of Ophthalmology*, followed by the journals *Ophthalmology* and *Investigative Ophthalmology Visual Science*. Although the journals primarily focused on ophthalmology have published numerous influential and highly regarded articles, the journals that focus on the broader medical field, like Nature and Lancet, for example, should not be dismissed. Despite having fewer publications in this topic area, these journals have a significant influence on the field of ophthalmology. The metric we provided should be useful for future authors who are interested in publishing articles in this field. Also, the bubble maps we provided for the last period should be useful in identifying the journals that actively published during this period.

The present bibliometric analysis provides a powerful set of tools and measures that provide insightful information about research output, impact, trends, and collaboration prospects, thus helping academics better understand this explored subject. One significant advantage of this investigation is its analytical approach. The subjectivity of the authors is not influenced by the use of quantitative tools, resulting in a more objective judgment. Furthermore, bibliometric analysis is cost-effective and reproducible, making it a practical and efficient tool. Another advantage of bibliometric analysis is the ability to study a substantial number of documents. This enables a thorough analysis of the topic area and a larger view of the research landscape. Moreover, the information systematized and classified according to certain parameters constitutes an important time-saving tool for authors during the pre-publication and research topic setting period, facilitating the selection of journals publishing on the desired topic, the most prolific articles as a starting point in identifying the current state of knowledge and unmet needs, as well as author collectives and nations publishing more often in the field for the creation or improvement of collaborative networks between authors interested in research in this field.

Although this field employs programs with a modern interface and efficient algorithms, it is crucial to recognize that there is still room for progress. The most remarkable advancement in this subject arises from the constant improvement of databases. As a result, better and more complete article indexing would result in significant advances in the field of bibliometric analysis. Another important feature contributing to its progress is the precise and accurate indexing of author names. The accurate indexing of authors would provide an accurate representation of their contribution to any given field and improve the identification of significant collaboration networks, leading to more valuable tools for future researchers.

## 5. Limitations

### 5.1. Molecular Docking Approach

The present in silico study has a number of limitations, such as its inability to reliably estimate the biological effects of a compound and its focus on a single static interaction between the ligand and the protein of interest, despite the fact that protein molecules are extremely flexible and adaptable in the biological milieu and may undergo significant conformational changes during compound binding.

Furthermore, the inability to assess the solvent effect may also have an impact on ligand binding. These limitations are noticeable at this initial phase of research investigation, yet they may be addressed through further studies in the future, such as the use of more advanced computational methods (e.g., molecular dynamics simulation, network pharmacology, etc.). Still, all findings obtained will require additional experimental validation prior to undergoing all the necessary steps for therapy approval.

Even though the most promising compounds selected and proposed for experimental endorsement based on binding affinity to ROCK1 or ROCK2 do not contain fluorine atoms, it is important to mention that among the first five results offered by SwissSimilarity’s digital platform are compounds containing fluorine atoms. The incorporation of fluorine atoms into compounds identified through ligand-based virtual screening poses challenges due to their high electronegativity and potential for forming strong protein interactions. Therefore, rigorous validation and optimization of the parent ligand and subsequent experimental investigations are imperative. While fluorine can enhance compound stability, its introduction can significantly influence metabolic behavior and toxicity, potentially altering a compound’s viability as a drug candidate. Some fluorinated substances may exhibit prolonged existence or distinct metabolic pathways, impacting safety profiles. Additionally, the presence of fluorine may intricately affect synthesis, demanding specialized reagents and conditions for fluorination reactions.

The present analysis proposes two candidates following the molecular docking study based on the highest potential binding to the target proteins impacting glaucoma, ROCK1 and ROCK2, representing a preliminary step in drug design studies, a step that needs to be confirmed by extensive computational studies (i.e., molecular dynamics, network pharmacology), in vivo in animal models, and clinical studies in different phases where efficacy and safety profiles are evaluated.

### 5.2. Bibliometric Analysis

The present bibliometric analysis, which emphasizes solely English articles evaluating treatments for ocular diseases, reveals a number of notable limitations. Primarily, the accuracy of the analysis may be limited by language bias, as the exclusion of papers written in languages other than English may exclude valuable perspectives and insights from various research communities.

In addition, by focusing exclusively on article-type publications, the analysis may have overlooked other valuable sources of data, such as case reports, books, and book chapters, which might offer supplementary approaches and knowledge on ocular disease therapeutics.

The focus on ocular disease therapies in the articles selected could additionally result in a low representation of broader studies investigating associated aspects, such as disease etiology, pathogenesis, and diagnostics. In the setting of ocular diseases, this may impede a comprehensive comprehension of the therapeutic landscape. Moreover, in the present bibliometric research, a large number of documents are included, and as a result, there may be some false positives among the initial results.

Lastly, bibliometric measures, such as citation counts, might not offer a comprehensive evaluation of the quality and influence of the articles that were selected. Variations in citation practices and the prominence of particular journals within the field may affect the perceived value of individual articles, thereby influencing the results of the bibliometric analysis.

## 6. Conclusions

The dual approach in this article proposes two compounds with possible application in the control of glaucoma conditions, but it is necessary that the results of this study be coupled with extensive computational assessments, in vitro experiments, in vivo validation investigations, and a bibliometric analysis focused on therapy, including laser-based therapy, for some of the most prevalent ocular pathologies, including glaucoma. These findings suggest that the parent compound ripasudil and the compounds identified via ligand-based virtual screening, ZINC000000022706 and ZINC000034800307, have similar binding patterns and affinity for ROCK1 and ROCK2. The compounds also showed a higher affinity for the proteins than fasudil, a known Rho kinase inhibitor. Due to the similarity in ligand–protein interactions and favorable pharmacokinetic properties, ZINC000000022706 and ZINC000034800307 should be further researched as potential ROCK1 and ROCK2 inhibitors. Additional research is necessary to evaluate their efficacy and safety profiles for possible future use as pharmacological agents, including experimental validations and a thorough study of their pharmacokinetic profile.

Examining publishing patterns found a constantly growing trend in published publications, indicating increased researcher interest and active involvement. Moreover, the United States has developed into the most prolific nation in this field, highlighting the region’s substantial investment in authors. China has shown a tremendous increase in interest over time, emphasizing its expanding prominence in the sector. The field of ophthalmology is developing more structured and defined collaboration networks. These findings provide useful insights into the dynamics of collaborative research and emphasize the necessity of encouraging interdisciplinary collaboration.

## Figures and Tables

**Table 1:** Five compounds with the greatest affinity for ROCK1.

Compound	Chemical Structure	Binding Affinity (kcal/mol)	Score of Similarity
ZINC000000022706	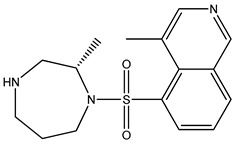	−9.0	0.772
ZINC000193357696	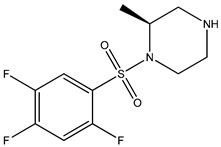	−7.3	0.508
ZINC000193358334	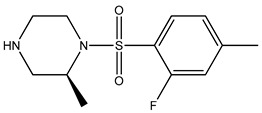	−7.3	0.492
ZINC000034800306	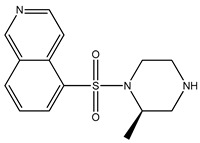	−7.1	0.590
ZINC000034800307	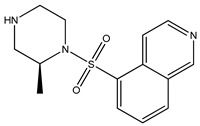	−7.1	0.590

**Table 2:** Five compounds with the greatest affinity for ROCK2.

Compound	Chemical Structure	Binding Affinity (kcal/mol)	Score of Similarity
ZINC000034800307	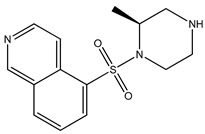	−8.8	0.590
ZINC000000022706	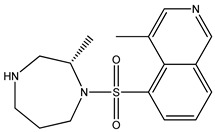	−8.6	0.772
ZINC000054371104	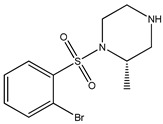	−7.7	0.492
ZINC000193357696	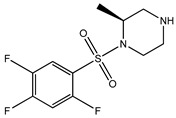	−7.6	0.590
ZINC000534634918	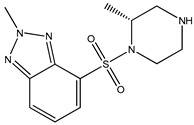	−7.6	0.590

**Table 3:** The findings of the computational ADME evaluation of possible Rho kinase inhibitors.

Characteristics	ZINC000000022706 (ROCK1)	ZINC000034800307 (ROCK2)
Formula	C_16_H_21_N_3_O_2_S	C_14_H_17_N_3_O_2_S
Molecular weight	319.42 g/mol	291.37 g/mol
Num. rotatable bonds	2	2
Num. H-bond acceptors	5	5
Num. H-bond donors	1	1
Log P	2.50	2.10
Gastrointestinal absorption	High	High
CYP2C19 inhibitor	No	No
CYP2C9 inhibitor	No	No
CYP2D6 inhibitor	Yes	No
CYP3A4 inhibitor	Yes	No
Lipinski	Yes	Yes

**Table 4:** Ten nations with the highest level of output and productivity.

Country	Papers	Citations	Average Citation/Article	Total Link Strength (TLS)
United States	6736	179,553	26.66	4451
China	2503	31,820	12.71	1151
England	1555	47,090	30.28	2482
Japan	1444	30,626	21.21	745
Germany	1432	39,223	27.39	2088
Italy	1188	25,012	21.05	1559
India	973	13,759	14.14	923
France	750	24,467	32.62	1572
Australia	739	22,583	30.56	1378
South Korea	713	12,330	17.29	354

**Table 5:** Top 10 prolific journals and their metrics.

Journals	No. of Papers	No. of Citations	Average No. of Citations per Article	IF	IF without Self-Citations	Publishing Entity
*Investigative Ophthalmology & Visual Science*	896	24,621	27.48	4.925	4.589	Assoc Research Vision Ophthalmology Inc., Rockville, MD, USA
*Retina—The Journal of Retinal and Vitreous Diseases*	799	17,913	22.42	3.975	3.617	Lippincott Williams & Wilkins, Philadelphia, PA, USA
*Graefe’s Archive for Clinical and Experimental Ophthalmology*	615	8208	13.35	3.535	3.372	Springer, Berlin/Heidelberg, Germany
*British Journal of Ophthalmology*	610	13,254	21.73	5.907	5.565	BMJ Publishing Group, London, UK
*American Journal of Ophthalmology*	601	19,127	31.83	5.488	5.128	Elsevier Science Inc., Amsterdam, The Netherlands
*Ophthalmology*	594	39,344	66.24	14.277	13.741	Elsevier Science Inc., Amsterdam, The Netherlands
*European Journal of Ophthalmology*	466	3257	6.99	1.922	1.743	Sage Publications Ltd., New York, NY, USA
*BMC Ophthalmology*	452	3712	8.21	2.086	1.992	BMC, London, UK
*Ophthalmology and Therapy*	415	1594	3.84	4.927	4.759	Springer Int Publ Ag, Cham, Switzerland
*PLoS ONE*	322	6701	20.81	3.752	3.608	Public Library Science, San Francisco, CA, USA

IF, impact factor.

**Table 6:** The most productive authors in the field between 2011 and 2023.

Authors’ Name	Latest Affiliation	Nation	No.	No. of Citations	Average Citations per Document
Bandello, F.	Vita-Salute San Raffaele University	Italy	112	2034	18.16
Hauswirth, W.W.	University of Florida	United States	92	4404	47.87
Maclaren, R.E.	University of Oxford	England	91	2916	32.04
Liu, Y.	-	-	85	1043	12.27
Zhao, M.W.	-	-	83	613	7.39
Chhablani, J.	University of Pittsburgh	United States	81	1172	14.47
Sahel, J.A.	National Institute of Health and Medical Research (Inserm)	France	76	3819	50.25
Shields, C.L.	Jefferson University	United States	75	2441	32.55
Freund, K.B.	Vitreous Retina Macula Consultants of New York	United States	73	3476	47.62
Holz, F.G.	University of Bonn	Germany	73	4809	65.88

**Table 7:** Top 10 most cited articles in the period 2011–2023.

Main Author (Year)	Title of the Paper	Scientific Periodical	IF	C	Ref.
Sawcer, S. (2011)	Genetic risk and a primary role for cell-mediated immune mechanisms in multiple sclerosis	*Nature*	69.504	1942	[31]
Heier, J.S. (2012)	Intravitreal Aflibercept (VEGF Trap-Eye) in Wet Age-related Macular Degeneration	*Ophthalmology*	14.277	1562	[32]
Martin, D.F. (2012)	Ranibizumab and Bevacizumab for Treatment of Neovascular Age-Related Macular Degeneration	*Ophthalmology*	14.277	1315	[33]
Okita, K. (2011)	A more efficient method to generate integration-free human iPS cells	*Nature Methods*	47.99	1298	[34]
Lim, L.S. (2012)	Age-related macular degeneration	*Lancet*	202.731	1216	[35]
Mintz-Hittner (2011)	Efficacy of Intravitreal Bevacizumab for Stage 3+Retinopathy of Prematurity.	*New England Journal of Medicine*	176.082	909	[36]
Quigley, H.A. (2011)	Glaucoma	*Lancet*	202.731	848	[37]
Mandai, M. (2017)	Autologous Induced Stem-Cell-Derived Retinal Cells for Macular Degeneration	*New England Journal of Medicine*	176.082	833	[38]
Tang, J. (2011)	Inflammation in diabetic retinopathy	*Progress In Retinal and Eye Research*	19.704	763	[39]
Rofagha, S. (2013)	Seven-Year Outcomes in Ranibizumab-Treated Patients in ANCHOR, MARINA, and HORIZON	*Ophthalmology*	14.277	701	[40]

C, number of citations; Ref, references.

**Table 8:** The most active organizations during the 2011–2023 period.

Affiliations	Record Count	% of 19,233
University of California System	885	4.60
University of London	812	4.22
University College London	749	3.89
Harvard University	608	3.16
Moorfields Eye Hospital NHS Foundation Trust	553	2.88
Johns Hopkins University	521	2.71
Harvard Medical School	515	2.68
Udice French Research Universities	456	2.37
University of Pennsylvania	389	2.02
Johns Hopkins Medicine	381	1.98

## Data Availability

All the information in the manuscript is supported by the mentioned references.
